# Minor Resistance: The Everyday Politics and Power Dynamics of Assistive Technology Adoption

**DOI:** 10.1145/3663547.3746465

**Published:** 2025-10-22

**Authors:** Stacy Hsueh, Danielle Van Dusen, Anat Caspi, Jennifer Mankoff

**Affiliations:** University of Washington, Seattle, Washington, USA; Occupational Therapist & Assistive Technology Specialist, Seattle, Washington, USA; University of Washington, Seattle, Washington, USA; University of Washington, Seattle, Washington, USA

**Keywords:** Assistive Technology, Power, Social Inequity, Minor Resistance, Empowerment, Intersectionality, Accessibility

## Abstract

In accessibility research, the choice to adopt or abandon assistive technologies (AT) is often treated as a stable proxy for functional fit: to adopt is to confirm a good fit between device features and individual needs, and to abandon is to signal poor fit. While useful for design, we argue that the framework is ill-equipped to account for the sociopolitical forces that shape AT use in historically underserved communities. In this paper, we propose a power-aware framework that recasts adoption not as a transparent expression of fit, but as situated negotiation of power. Drawing from an eight-month ethnographic study at a local nonprofit, we examine how low-income, racially-diverse, and disabled families navigate institutional cultures that reinforce normative expectations around disability and AT use. Building on postcolonial theories of power, we introduce the concept of minor resistance to describe the subtle, everyday tactics through which individuals lower the cost of access on their own terms. We argue that this shift in analytical lens reframes the goal of accessibility design from optimizing use to lowering the cost of choice. We conclude with implications for how designers can support community-driven responses to structural barriers by centering self-determination.

## 1 INTRODUCTION

For more than two decades, accessibility researchers have been interested in facilitating successful adoption of assistive technology (AT) used by people with disabilities [47, 74, 81]. In popular discourse, AT is figured as a technology of empowerment: “For most of us, technology makes things easier. For a person with a disability, it makes things possible.”, says disability rights activist Judy Heumann. From audio notetakers to tactile displays, AT has enabled a wide range of activities and is used extensively by people with disabilities. It is thus no surprise that a significant body of accessibility literature is dedicated to understanding barriers to AT adoption and factors for AT abandonment [47, 62, 74, 81] as well as removing barriers that stand in the way of use, by making ATs more flexible to customization [21] or more usable [25]. On close examination, the most enduring narratives across these works usually involve improving the “fit” between technologies and the diverse needs of users. Within this framework, adoption is treated as a proxy for fit: to adopt is to confirm good fit, and to abandon is to reveal poor fit.

This emphasis on fit frames empowerment as a technical problem – a state that can be arrived at by fine-tuning the match between device features and user needs. However, such view obscures the contextual factors that influence how technologies are used and experienced. As researchers have shown, promises of empowerment depend heavily on one’s socioeconomic conditions and often fall short for historically underserved communities [5]. In fact, people will use an AT even when it is unusable, because they are structurally compelled to switch to a new device [59, 72]. There is also growing evidence that the process of seeking or adopting accommodations can itself be burdensome or harmful [65, 83], suggesting that non-use is a legitimate and strategic response to structural tensions. These burdens are often more pronounced for historically underserved communities, who are subject to structural inequities that amplify the cost of access, through stigmatization, administrative gatekeeping, or prior experiences of harm.

Much of the critique in this area suggests a need to go beyond current model of adoption-abandonment as binary, stable indicators of fit, as it is ill-equipped to capture the day-to-day realities that underserved communities grapple with. It assumes that use is always desirable, that non-use is a problem to fix, and that the choice is freely made. Instead, taking cues from Liboiron’s notion of *constrained agency* [49], we propose to view choice not as a transparent expression of fit, but as situated negotiations of power – a response to precarity, institutional demands, and/or past harms. Specifically, we argue that we need a framework that tackles accessibility as a question of power, in addition to a question of design. What this means is that instead of treating AT as devices existing in a vacuum [4], we see it as existing at the intersection of social practices, technological affordances, and institutions – a sociotechnical assemblage [23] – and thus cannot be analyzed independently from them. This implies that use is shaped through balances of power.

To analyze the role of power in AT use, we draw on sociological studies of everyday forms of resistance [76] to understand how families navigate and resist the structural conditions that shape access. In particular, we propose a new way of thinking about designing for and with underserved communities: instead of optimizing use, we ought to lower the cost of choice. To that end, we introduce the concept of *minor resistance*: subtle, often improvised strategies through which individuals manage the cost of access-related choices in their daily lives. By foregrounding minor resistance, we aim to shift the analytical focus of accessibility research: from what technologies can be made to do (i.e. top-down interventions), to how they might support and amplify the existing ways people assert agency within disempowering systems (i.e. bottom-up strategies).

We begin our exploration by first situating discussion of power and resistance within feminist and critical race theoretical scholarship of power and resistance, unpacking different notions of empowerment in accessibility research along the way. We then articulate the tensions caused by current approaches to AT adoption and abandonment in historically underserved communities. This paper makes the following contributions:

- We provide an analysis of how power works in everyday AT use in underserved communities.
- We introduce the concept of *minor resistance* to describe the various forms of resistance in reaction to the frictions that inevitably arise when families encounter rigid structures.
- We offer critical questions for accessibility researchers for thinking through power and states of precarity in AT contexts.
- We offer implications of minor resistance for reorienting design practice around community-driven approaches to accessibility.

## 2 THEORETICAL BACKGROUND

In this section, we will give an overview of the theories that are grounding this paper. Specifically, we will identify the theoretical frameworks which helped us explore power dynamics and empowerment as theorized by Jo Rowlands [68], the derivation of “minor resistance” literature from everyday forms of resistance.

### 2.1 Putting Power Back in Accessibility

Empowerment is often invoked in accessibility literature without much articulation of what *power* is. Rowlands, in her examination of empowerment [68], emphasizes that understanding power dynamics is crucial for any discussion of empowerment. She highlights that true empowerment must engage meaningfully with power structures that shape our societies, recognizing that power is not just about individual capacity but also about the systemic forces that enable or constrain it. For empowerment to be meaningful, we must first develop a holistic understanding of what power is.

One way to understand power is through its four instantiations, first conceptualized by Rowlands [69], and subsequently taken up by numerous scholarship such as [31, 87]. The first type, *power-over*, refers to the traditional concept of power as domination and control. This form of power is often seen in hierarchical structures where one group or individual exerts influence over others, leading to oppression and inequality. In contrast, *power-to* focuses on the capacity to act and bring about change in response to forms of oppression. *Power-within* is related to a person’s sense of self-worth, whereas *power with* emphasizes that power grows out of collaboration and relationships.

In AT literature, *empowerment* typically refers to enabling users to create AT for themselves [14, 39, 64], increasing independence [52, 77], and improving performance in daily tasks [29]. This rendering of empowerment emphasizes self-sufficiency and self-reliance. Complementing it, researchers have proposed an alternative view of empowerment rooted in interdependence [10, 88], which describes the ways access is created through “relationships between people, ATs, and environments”. These two distinct renderings of empowerment are also picked up by Parent [61]. Viewing these forms of empowerment through Rowlands’ lens of power, we see that independence framing can be mapped to *power within*, which is an individual’s ability to achieve empowerment on their own, whereas the interdependence framing maps to *power with*, which refers to the shared power that grows out of collaboration and relationships. The notion of minor resistance introduced in this paper can be seen as a third form of empowerment, *power-to*, which names the the “unique potential of every person to shape their world”.

In this paper, we explore power-to as a frame for designing ATs that support people in self-assertion against systems of power. Examples of people rebelling against hostile systems or structures abound in disability communities. Consider, for example, activists in London who poured cement on “anti-homeless” spikes installed by a supermarket to curb “antisocial behaviors” such as smoking [63]. Artist and design researcher Sara Hendren would call this a form of design activism which “uses the language of design to create political debate” [36]. We want to build on and extend existing efforts of design activism by drawing on lessons of minor resistance we have observed in our study. We see this design practice as (1) helping people live with, resist, and subvert the inflexible systems and (2) helping researchers recognize, understand, and support minor resistance as a key to sharing power in design.

### 2.2 “Weapons of the Weak”: Conceptualizing Minor Resistance

The idea of popular resistance, as explored in sociology and anthropology, offers valuable insights into actions that might be read as resistance to AT adoption. A key reference is James Scott’s definition of everyday resistance, which he describes as a “weapon of the weak” [76]. He characterizes it as “an integral part of the small arsenal of relatively powerless groups” [76], manifesting in behaviors such as *foot-dragging*, *feigned ignorance*, or *sabotage*. Scott argues that political scientists have often neglected the extensive range of political actions undertaken by marginalized groups, as these actions tend to be more subtle and thus invisible. He challenges the traditional narrative that portrays vulnerable groups as apathetic or too overwhelmed to engage politically, illustrating instead how they participate in politics through everyday acts of resistance aimed at challenging authority and reclaiming autonomy [76]. In Scott’s perspective, these everyday forms of resistance serve as vital means for marginalized groups to express their political voice [76].

Our notion of *minor resistance* weaves together Scott’s concept of everyday resistance and recent interdisciplinary studies of resistance that views it as an oppositional act deeply intertwined with various, intersectional power dynamics. This resistance can manifest in forms that range from covert actions to organized movements [8, 51]. Resistance unfolds “in a process of social interaction” [42] involving three key players: (1) the individuals engaging in resistance (in our study, the disabled communities), (2) the agents of power (in our study, the insurance companies, hospitals, and school districts), and (3) various observers (other stakeholders such as the non-profit in our study). This definition emphasizes the roles of power and agency. We understand power as operating on three levels: “muscle power”, “agenda-setting power”, and “discursive power” [53], while agency is conceptualized as the ‘power-to’ [87].

## 3 RELATED WORK

### 3.1 Pitfalls of AT Adoption and Abandonment Narratives

Research on AT adoption and abandonment spans multiple disciplines – from HCI [93] to education [7, 56], psychology [15, 75], and occupational therapy [20, 33] – each foregrounding different explanations for why ATs are taken up or discarded. In HCI and accessibility research, two dominant frameworks have shaped how these dynamics are understood: the Technology Acceptance Model (TAM) and the Matching Person and Technology (MPT) model. TAM, developed by Davis [22], conceptualizes adoption as a function of “perceived usefulness”, “perceived ease of use”, and “behavioral intention”. It has been widely used to examine design factors that influence adoption, such as in Djamasbi et al.’s [28] study of web accessibility for blind users or Franz et al.’s [30] work on mobile accessibility for older adults. The MPT model, developed by Marcia Scherer [73], describes a person-centered approach that emphasizes that successful AT adoption depends on how well a device aligns with an individual’s needs, values, and social contexts. Abandonment occurs when there is a mismatch between the person and the broader environment in which the device is used. This framework has influenced a range of HCI work that examines the social dimensions of AT use. For example, Shinohara & Wobbrock [81] highlight how social stigma about disability influence AT use, particularly in shared or public spaces. Together, TAM and MPT provide complementary and overlapping lenses for understanding adoption: one that centers usability and user intention, and the other that emphasizes the fit between technology’s functions and features and the users’ goals and values.

However, looking at technology use through the lens of adoption and abandonment tends to filter out stories of everyday creative work people exercise when encountering inaccessible ATs. For example, Kane et al. [43] found that individuals with visual and motor disabilities conceive various strategies to adapt inaccessible devices for their purposes. Similarly, Borodin et al. [12], in their study of screen reader users, documents a range of adaptive strategies for navigating inaccessible web. Jacobson [41] examines how individuals customize assistive devices not only for practical reasons but also to manage social stigma surrounding the use of AT. These studies suggest that the line between adoption and abandonment is far more fluid than traditional models suggest, shaped by user modifications and workarounds.

Researchers have begun to challenge adoption and abandonment rates as metric of success or sign of failure. Dawe [24], for example, questioned the usefulness of having single adoption rate for all AT, pointing out that such metric obscure “large differences among the devices and the populations that use them”. Not only does adoption rate lack analytical granularity, it also “fails to reveal the ways and contexts in which technology is used”. Taking this critique further, more recent work [82] advocates for a shift in how abandonment is understood in clinical contexts: rather than interpreting non-use of AT as abandonment, they suggest recognizing it as a deliberate choice made by individuals and families “to use a full range of multimodal communication [that better] meet[s] their own needs”. Similarly, Seymour [78] shifts attention from seeking solutions for *why* people abandon AT to “the values and beliefs of disabled people as they choose to engage with or to abandon technology”. These works recast abandonment not as problem to be solved, but as a lens through which to better understand the deeper motivations and everyday lived realities that shape their decision to use or not use AT. However, relatively little is yet known about how disabled communities in under-resourced settings navigate these choices.

### 3.2 AT Use in Historically Underserved Communities

A growing body of literature in HCI and Accessibility research is concerned with what it means to design AT for and with historically underserved populations. These communities often face systemic barriers that limit access to resources such as healthcare and education. These barriers include economic constraints, limited technological infrastructure, and low digital literacy, which collectively hinder the adoption and effective use of AT. Addressing these challenges requires a nuanced understanding of the unique needs and contexts of these populations.

Yusif et al. [93] have studied the barriers to adoption in older adults, highlighting issues such as physical limitations, lack of digital skills, and socioeconomic factors that hinder the use of AT. Similarly, scholars have studied social media use among low-income blind people in India [86], revealing how these individuals navigate and utilize digital platforms despite significant accessibility challenges. Research in the Global South [70] has also shown that intermediated technology use, where individuals rely on others to access digital services, is a common practice among underserved populations.

While this work highlights critical access barriers, less attention has been paid to how power (manifested through institutional structures or gatekeeping practices) operates in these contexts: how it shapes what resources are available, but also constraining the range of choices communities can meaningfully make within systems not built for them.

### 3.3 AT Through a Sociotechnical Lens

HCI scholars, accessibility researchers, and critical designers have examined the politics of assistive technologies, from AI that “sees” [11], to prosthetics [37], cochlear implants [79], and disability dongles [40]. These works show how technoableism [80] sees disability as a problem to solve and sees technology as enabling a “return” to normative ways of being. The AT’s that have shown up in our study can also advance particular worldviews. For example, most communication devices assume English as the primary language of communication, and since many families in our study speak little to no English, they might choose not to integrate these devices into their daily routines.

While computational artifacts can embody and perpetuate specific worldviews [18, 91], AT extends beyond devices. According to Administration for Community Living (ACL), AT also includes “services necessary to get and use the devices, including assessment, customization, repair, and training” [1]. In HCI and computing research, we tend to have an device-level view of AT, but shifting to a service-level view – such as the ecosystems of care around 3D printed AT [71] – lets us see that devices do not exist in the vacuum. They work as part of a larger sociotechnical system. So beyond biased notions about disability and bodily norms manifesting through design features (or lack thereof), they can also perpetuate through the process by which the device is acquired. Speech language pathology literature discusses how AT use is inseparable from medical diagnoses and assessments, and healthcare professionals can often get things wrong, such as over-identifying non-English speakers as language disordered because there are no resources to test them in their own language [13, 19, 60]. Communications and disability studies scholar Meryl Alper notes in her book *Giving Voice* [5] that AT such as the iPad and mobile app Proloquo2Go can embed structural inequalities: the institutional ecosystems that bolster the use of those technologies often privilege families of a specific socioeconomic status and marginalize working-class and low-income families. She advocates studying AT as a sociotechnical practice, challenging claims of empowerment and making visible the role of culture and policy in shaping AT use.

While this work reveals how structural inequities are embedded in AT design and provision, less is known about how families on the margins actively navigate, adapt to, and resist these various biases, crafting strategies to assert agency within, around, and against them.

## 4 METHODOLOGY

To understand how disabled families in underserved communities navigate and negotiate AT systems, we conducted an ethnographic field study at a local non-profit that offers AT support services to low-income and racially-diverse communities.

### 4.1 Data Collection

We conducted ethnographic fieldwork spanning eight months (from February 2024 to September 2024), including a wide range of interviews and ethnographic observations with the staff at Open Doors for Multicultural Families, a Seattle-based non-profit organization that provides assistive technology support and other supportive services for families with disabilities from diverse cultural backgrounds. To contextualize this empirical work within the broader ecosystem of AT practices and infrastructures, we complemented interviews and ethnographic material with a collection of assistive technology webinars and additional archival resources described below.

#### Research site:

Open Doors for Multicultural Families (ODMF) is a non-profit organization that help families with disabilities navigate disability services and in particular get access to assistive technologies. The families served are primarily immigrants, refugees, and people of color who come from diverse cultural communities such as African American, African immigrant, Asian, Southeast Asian, Hispanic/Latinx, Middle Eastern, and Eastern European. 89–90% of the families served come from low- to very low-income households and may have limited English proficiency. ODMF provides services across the lifespan, designing programs for infants and young children to adults and seniors. Their AT program consists of an AT lending library that offers a range of low-tech to high-tech assistive devices.

##### 4.1.1 Observations and ethnographic fieldnotes.

Our ethnographic fieldnotes were developed from first-hand accounts of case management, assistive technology consultation sessions, workshops, and lending library inventories collected over 7 months by the first author as they worked at ODMF as an intern.

During these months, the lead author spent 5–10 hours a week attending and observing workshops (e.g. making DIY AT, Open House, and navigating AT services), school visit to a local high school, consultation sessions, Friendship Circle, information sessions, and staff meetings. They also helped with doing inventories for the lending library and researching new assistive devices to add to the library.

As part of this fieldwork, 12 AT consultation sessions were observed. In these sessions, the AT specialist (second author), sometimes alongside case managers, engaged families in interviews to assess their needs and recommend suitable devices for short-term loan from ODMF’s AT lending library. Detailed fieldnotes were taken both during and immediately following each session to capture interactions.

##### 4.1.2 Interviews with case managers and families.

We conducted interviews with case managers and clients at Open Doors. This work included five interviews with case managers, three interviews with program managers, 13 interviews with families who are enrolled in the AT program at Open Doors, and one interview with a speech language pathologist (SLP). Each interview averages 90 minutes in length (ranging from from 60–110 minutes each). We complement the 22 interviews with extensive conversations over 6 months with an AT specialist at Open Doors and another intern during which detailed field notes were taken by the first author, along with other informal conversations with ODMF staff.

We interviewed families who are currently enrolled in the AT program at Open Doors. Each family included at least one parent (with or without disability) and a child with disability. In the majority of cases (12 families), it was the parent seeking services for their child (the children’s ages range from 3 to 22 years old, with 3 being the most common age among participants). The disabilities represented in the sample included cognitive disabilities (10x, predominantly non-verbal autism), mobility impairments (2x), and hearing impairment (1x). For all the interviews, we spoke with the parent. However, in two interviews, the child was also present. During these interviews, we would speak with the parent primarily and ask the child simple yes/no questions, with the child providing responses through a communication device. For six of the participants whose primary language is Spanish, we also used LanguageLine for interpretation. LanguageLine provides phone interpreting and is the main service used at Open Doors.

For the participant coding scheme, we labeled case managers as CMx, program managers as PMx, families labeled as Px, and speech pathologist as SLP.

##### 4.1.3 Documents and webinars on cultural AT and DIY AT.

We collected archival material based on resources shared by Open Doors staff. We focused on resources concerning assistive technology services. The items we engaged with included articles, webinars, and podcasts. In focusing on industry understanding of AT, we were interested in understanding what happens when AT interacts with policies and travel through government agencies in order to draw a more accurate landscape of the on-the-ground realities of AT use. While we did not formally analyze these materials, they served as important contextual resources that informed our understanding of dominant discourses around AT access, policy, and practice.

### 4.2 Data Analysis

We analyzed our data thematically using inductive methods informed by a contextualized approach to grounded theory [16]. This approach helped us surface power and resistance as central threads woven through the different narratives.

We developed reflexive memos based on our field notes, interviews, and other empirical materials. These memos were reviewed together during weekly meetings, where we collaboratively generated initial codes and observations. Emergent themes included institutional logics embedded in AT services, tensions between institutional clocks and lived timelines. Through iterative rounds of analysis, we refined our interpretations, culminating in our focus on minor forms of resistance and generative frictions that occur at the intersection of institutional expectations and lived experiences.

To develop a power-aware lens to investigating technology use, we analyzed (1) the various sites and mechanisms through which power is exercised along the AT provision pipeline, and (2) everyday acts of resistance families employ to asser their selfhood, values, and temporalities. We refer to these acts as *minor resistance* – a form of counter-politics that operate not through overt protest but through subtle, often improvised tactics of survival. These practices reflect what Sara Ahmed describes as “willfulness” [3]: the friction that occurs when people push back against systems no built with them in mind.

### 4.3 Positionality

The collection and analysis of data are influenced by our experiences as accessibility researchers and practitioners. The second author brings professional field experience as an AT specialist, working in AT provision. Three of the authors also have experience with caring for family members and interacting with many of the same systems as the families from our study. As a team, we hold varied relationships to race, class, and disability. While none of us shares the full set of social positions held by the families in this study, all of the authors are first- and second-generation immigrants, three are multilingual, and two are parents of disabled children. Although we are in positions of relative privilege as researchers and practitioners within U.S. institutions, we also contend with structural barriers that surfaced in our fieldwork. Throughout this work, we remain attentive to our solidarities and differences with the families we engaged with. We also place strong emphasis on grounding our empirical results in relevant theoretical frameworks [35], particularly drawing from postcolonial studies of resistance to understand agency is enacted in response to top-down processes.

## 5 RESULTS

In this section, we present minor resistance and position it in a broader landscape of power dynamics. We then identify four types of minor resistance.

While our findings center on schools, hospitals, and insurance companies as the proximate sites through which families navigate AT access, these institutions do not operate in isolation. They are embedded within a broader U.S. sociopolitical landscape marked by racialized rhetoric, austerity politics, and escalating Big Tech and state power. Though a full accounting of these forces lies beyond the scope of our current study, we see this context as critical for situating the more immediate power relations described in the following section.

### 5.1 Framing Minor Resistance: Power Situations in Everyday Disabled Lifeworlds

**Figure 1:**
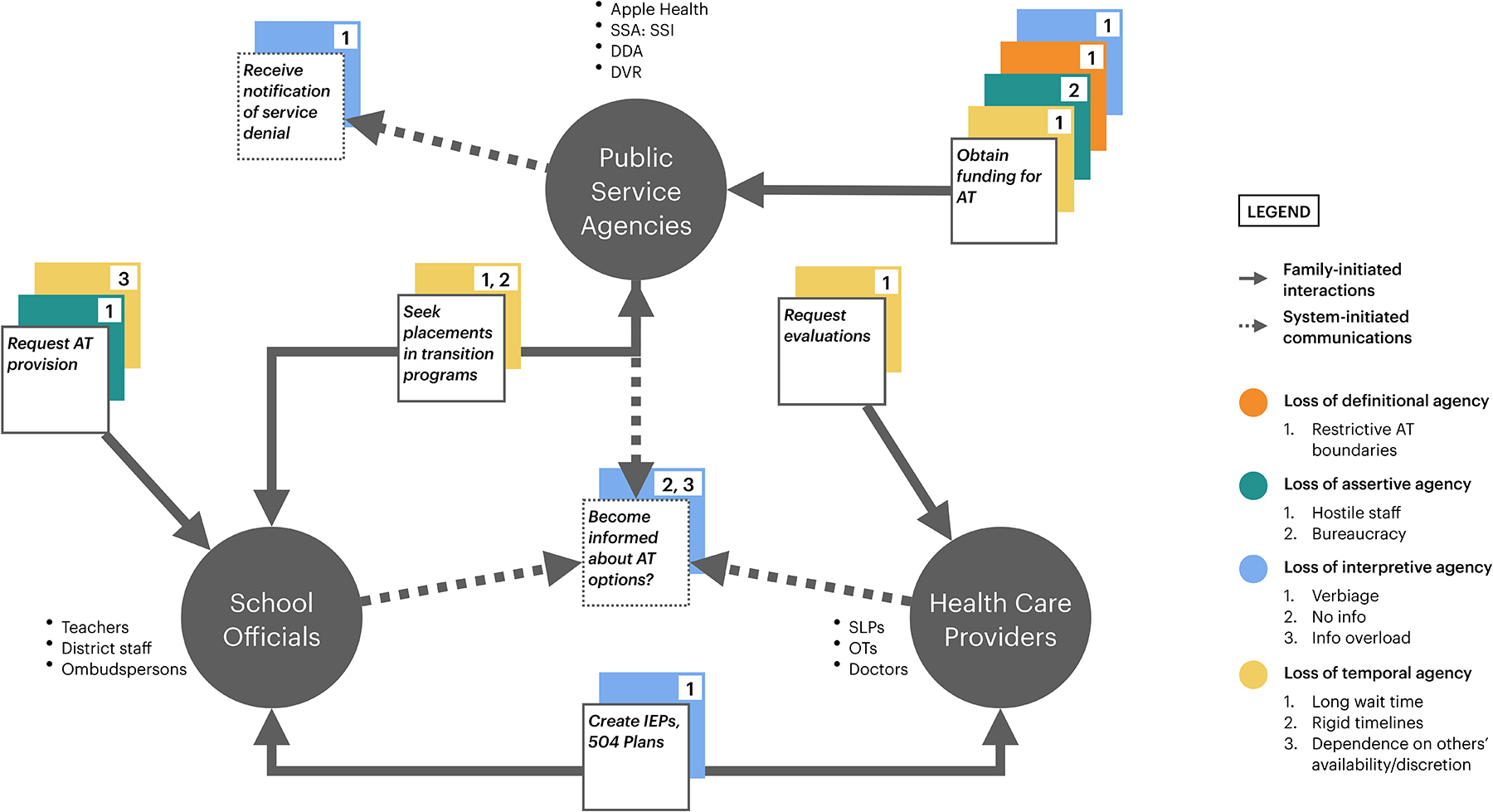
Visualization of how interactions with key institutions (schools, hospitals, and insurance companies) lead to different forms of agency loss among families. These include losses of definitional, assertive, temporal, and interpretive agency.

Before we turn to specific practices of minor resistance in the next section, we provide here an understanding of the cross-cutting systemic factors that can influence a family’s adoption of AT. Specifically, we show how power works through these systemic factors to impact access to AT and how families’ voices are heard (see Figure 1).

#### 5.1.1 Loss of definitional agency: regulation of AT boundaries.

The families we interviewed regularly engage with healthcare providers, insurance companies, and school districts to obtain funding for assistive devices. In practice, these organizations serve as gatekeepers, determining what qualifies as AT through evaluation processes that are biased towards the familiar. This creates a tiered effect [65]: the families with more common, recognized access needs have greater access to AT, while those with less predictable needs are left to figure out solutions on their own. Very often, the real needs of these families are in direct conflict with the various narrow definitions of AT used by these organizations.

For example, some ATs that are designed for children can be viewed as just “toys” to industry providers who do not have the knowledge and expertise of how these tools can be used to help regulate the nervous systems of children with disabilities. An example of one of these “toys” is the spin disc, which is a platform that a child can sit on to spin around. As emphasized by the AT specialist and CM4, spinning activates the vestibular system and are particularly useful for neurodivergent children in helping them get ready for bed or winding down after a stressful day at school.

Case manager CM4 described a time when she tried to help a child with autism to receive financial support for a sensory sit-n-spin seat:

“I have this one family…their child likes to be on the floor all the time…[the family wanted buy a spin disc], but it’s $250. The parents couldn’t buy it. We couldn’t buy it on my department because **it looks like a toy**. But it’s not [a toy]. It’s a spin disc. And they’re like, No, we cannot buy…got denied many times.”

The “toy” designation extends to other therapeutic items as well:

“Like weighted pillow. It’s to give the kids a sense of security…When you put it on your legs, it makes you feel your legs. It helps you stay sitting for longer periods. However, they will not see it as an item that the child needs. They see it as a toy. The bouncing ball too. The bouncing ball helps the kids who have motor needs, like their motor skills are not where they’re supposed to be. I was like, no there has to be a way that you guys can buy it and give it to the families.”

(CM4)

Certain educational items are also considered “toys” even though they play an important role in a non-verbal child’s curriculum:

“Some educational materials too. Like I was trying to give one of my families a [talking flashcards] tablet, where you put a card on the back, and it will tell you, say, an apple. It’s amazing. [And when I tried to get them] for my families, they said no that’s a toy. I said it’s educational. No, it’s a toy.”

(CM4)

Other times, it can be tricky to receive financial support for AT because the *boundaries* of AT is not clear:

“An iPad, they won’t buy it…because it doesn’t come with the applications [installed] to use it as a communication device. [Families] have to buy the applications separately, right? And a lot of the applications you have to pay monthly fees. So it’s not something that you can just download to your device and you’re done. So we will not buy a tablet for the families. We will not buy the application, either, [even] if they already have a tablet.”

(CM4)

The iPad does not become an AT until it has specific communication applications installed, even though it is the industry standard for non-verbal children to communicate with others outside of analog picture cards.

These competing criteria for what counts and does not count as AT make eligibility for AT an elusive pursuit for low-income families. They are consistently at the mercy of the insurance companies and other funding agencies who set the terms of inclusion for AT support, and by extension, defines the typical individual who qualifies to receive support.

#### 5.1.2 Loss of assertive agency: constant flow of judgment.

In navigating various services, families frequently face a steady stream of judgment, dismissal, and bureaucratic inertia that create feelings of anxiety, dread, and unease. Over time, this emotional toll erodes their confidence and willingness to voice or advocate for their needs, leading many to internalize rejection as inevitable.

Interactions with institutions can incur a great deal of emotional cost for the families. They are often forced to navigate a system that does not acknowledge their expertise in understanding their own or their children’s needs. In this context, institutions can silence families by framing their requests as burdensome or troublesome, which creates an emotionally taxing process. The violence in this dynamic is quiet, often manifesting in the slow wearing down of families’ resolve and their ability to advocate for their children. As a result, parents are left feeling powerless, with a fearful orientation to navigating the system, unsure if their voices will ever be truly heard or if they will simply be exhausted into compliance.

When applying for AT support, families are often subjected to experiences that are demoralizing and stressful. For example, they can get asked intrusive questions:

“A lot of people that work at SSI, they don’t care about you or your child, and they don’t care about your feelings, they can be very mean…they ask questions like, ‘What do you need the money for,’ ‘What are you gonna do with this,’ ‘Do you really need to do this and that?’…A lot of parents are like, ‘They’ve made me feel like I’m taking money from their paycheck’…and they make me feel humiliated.”

(CM4)

At school, parents can also face repeated rejections of accommodation requests for their child, such as a tablet for classroom communication, even when such accommodations are legally mandated. CM4 mentions IEP meetings where the psychologist and the special education teacher will say, “*I don’t get paid enough for this*”, preferring to convince the parents to bring their own tablet than to fill out all the paperwork needed to acquire one for the classroom. This hostility is felt by the parents:

“But a lot of my parents, they will start crying. Because if the [teachers] feel like this, and they say things like that in front of me, hat did they do when I’m not there? What did they do with my child? Do they care about my child? Do they treat my kid differently than other kids? What did they do? So it’s very upsetting, walking into a meeting like that when [the teachers are] not happy with what they’re doing.”

(CM4)

Through these experiences, the families feel dismissed as nuisance, their dignity shattered in the process. This contributes toward a fear-based relationship toward help. Many times, the parents just want to give up:

“A lot of the parents get discouraged. They just want to give up. They want to be done and to try something different. They don’t want to try for what they’ve been waiting for. It has happened with clients where they’re waiting for a communication device for AbleNet. And a lot of families are like ‘**I’m done. If it doesn’t happen, good**.’”

(CM4)

These persistent experiences of rejection and humiliation shapes the families’ willingness to fight. Instead of reaching out to inquire about the status of their benefits application, for example, families often just assume they do not qualify and do nothing. After all, it is hard to keep fighting when every effort feels like a losing battle.

#### 5.1.3 Loss of interpretive agency: information opacity and asymmetry.

Institutions often operate in ways that are opaque and complicated, which significantly affects families’ relationships to AT. When decisions about what families should access or know are made by institutions, it strips away their agency. This lack of transparency creates a power imbalance where institutions control what information is shared and how it is presented, leaving families with little say in what they are allowed to understand or pursue. As a result, families often find themselves navigating a maze of bureaucracy and jargon, unable to make fully informed choices about AT options for their loved ones. This loss of choice in what to know diminishes their sense of control, further entrenching the barriers to using and adopting AT effectively.

One such form of opacity has to do with the knowledge required to articulate needs. Obtaining the right AT is not merely a matter of access. It also depends on knowing which device is appropriate for a specific situation. For many families, the tools and supports needed to make these determinations simply do not exist.

Epistemic gaps emerge on multiple levels. Beyond language barriers, families often lack clear pathways to reliable information. Many enter meetings with case managers unsure of what to ask for, or even what kinds of support might be available or helpful to them.

These epistemic gaps appear on multiple levels. Beyond language barriers, families often lack clear pathways to information. Many enter meetings with case managers unsure of what to ask for, or even what kinds of support might be helpful to them:

“To be honest, I didn’t know what to expect. I really didn’t have expectations. I was looking for help because I was feeling kind of lost. And I was desperate. I didn’t know where to start. And that’s how I started. I actually asked, ‘You know, well, what resources do you have? How can you help me? This is what I’m struggling with’. My daughter has just recently been diagnosed with autism. So that’s how I started finding out about the playgroups, about the meetings, about what [she] needed to go to college, about technology. I didn’t have anything at that time.”

(P6)

Although the absence of interpreters during school or medical visits can create significant barriers for non-English-speaking parents, translation gaps often persist even in the presence of interpreters:

“I always ask them, ‘Do you understand what they mean by that?’ Even if you have an interpreter and [they] translate words exactly how the other person said it, that doesn’t mean you understand what they’re saying. Because if you have not been in the system, and you’re new to this, you don’t know a lot of the terms that they use.”

(CM4)

In short, understanding the system is different from speaking the language – the system has its own vernacular. As CM2 puts it, “*language access shouldn’t just be for people that are, you know, English as a second language. It needs to be accessible for people on all educational levels*”. Language can become a barrier for both English and non-English speakers, not only in translation, but in how information is communicated:

“The way that verbiage is put in the language, I would say, in as far as written terms and conditions and pro cesses and the way that is presented, can be spoken above someone’s head…above their educational level, above the vocabulary that they’ve been introduced to, you know, above their glossary?”

(CM2)

In many cases, families are denied services without understanding why (“*I did it exactly how I understood it, you know?*” (CM2)) Often, they only discover afterwards that forms need to be filled out differently, in ways that are not specified explicitly on the form. As CM1 articulated, “*the biggest challenge is awareness…the difficulty is really about the inability to ask the right questions*”

As a result of these translation gaps, the systems designed to support families remain opaque to them. Although there is a wealth of information about AT support, there are limited infrastructures to help families navigate these complex systems. The information they receive are fragmented and incomplete, at best. Far too often, the families are unaware of AT’s that could meet their child’s specific needs such as Tobii DynaVox, a communication device operated through eye-tracking. CM1 wonders: “*Who’s the first one responsible of communicating the existence of the device and its functionality to the family?*” Is it the specialist, the family doctor, or the insurance companies (noting that insurance companies have historically been uninformed about certain benefits)? While useful information sometimes reaches families, they are often delivered half-heartedly:

“I would say the help that we’ve received from the school is good but it was limited let’s say…’Your daughter needs to find some therapists.’ They only gave me the information, but the rest is completely up to me to contact [people] to find the solution”

(P11)

In many school districts, a single AT specialist is responsible for multiple schools across the district. Resources are insufficient for one person to manage all students with IEPs and their AT needs. In these processes, acquiring the right AT feels like a chance game.

Opacity also manifests in case managers’ decisions to simplify the complexities of navigating the system. For example, the AT specialist shared a story about a DDA case manager assisting a Dari-speaking family in obtaining an iPad with a communication app for their non-verbal autistic son. The process was complex and fragmented, involving multiple stakeholders and separate applications, each with its own timeline and service provider. To avoid overwhelming the family, the case manager intentionally withheld system-level details, such as the need to navigate multiple independently operating services. Although this approach aimed to reduce stress, the resulting lack of transparency ultimately deepened the family’s confusion and sense of powerlessness. After five months of waiting with minimal information or updates, the AT specialist intervened to seek clarification and follow-up. Without this advocacy, the family may have abandoned the process altogether.

#### 5.1.4 Loss of temporal agency: (re)production of chrononormativity.

Families often have little control over their own timelines, since external forces – like the pace set by healthcare providers, funding cycles, and bureaucratic processes – follow rigid, predefined clocks. These systems dictate the “when” of AT access: when a device is deemed appropriate, when funding is approved, when referrals are processed, and when training is scheduled. Rarely do these institutional rhythms align with the developmental needs, daily routines, or embodied experiences of disabled children and their families.

This loss of temporal agency – the ability to determine the pace at which one learns, adapts, or decides – can lead to a deep sense of powerlessness. One mother (P6) described calling every organization listed in a folder her doctor had given her, only to be met with months of silence and long waitlists. During those months of waiting, her daughter’s condition steadily worsened. Other parents similarly described being trapped in limbo by diagnosis-based access policies, forced to wait for the “right” referral before becoming eligible for devices, even when they already knew what would help their child.

Practitioners, too, are caught in the pressures of institutional time. One SLP noted that applying for device funding required unpaid hours of documentation and continuing education, time they simply do not have under current billing codes: “*Getting a device funded requires a lot of written documentation, time that we’re not paid for and can’t bill insurance for, based on the way our codes are built up…it also involves all of …the hours I spend…understanding the process, being up-to-date with funders and the application process. So most SLPs…even if they wanted the knowledge, maybe don’t have the time, or don’t see it as being cost effective*.” PM3 echoed the time balance point from the perspective of teachers, describing that they might be hesitant to install a high-tech communication assistive device because of the time it takes to “*implement the system…and download the right images*”, time that they may not have.

These temporal frictions are deeply consequential. For families, delayed access to AT can stall development during critical periods, especially for children already experiencing delays. P4, describes her son with severe autism who missed learning key skills in school. Now 22, he is too old to return to the regular curriculum and has aged out of after-school programs, leaving him in an educational standstill. Even when families find potential programs, waitlists can stretch so long that professionals themselves warn not to bother applying. As one mother (P9) puts it: “*They let me know that it’s too long. They are honest with me – they don’t want to make me wait for long*”.

Each institution – schools, hospitals, insurance companies – has its own chrononormative expectations that dictate how a body ought to operate and progress over time. And bodies that move out of step with these institutional clocks are left behind. In such cases, AT use is inextricably constrained by a misalignment between lived time and institutional time, a temporal mismatch that could discipline bodies into failure.

### 5.2 Asserting Agency Through Minor Resistance

In the previous section, we conceptualize power as efforts to enforce norms, bodily or otherwise, through standards, emotions, opacity, timetables. We have described the different ways families might find themselves coming up against rigid structures and how those structures could act as instruments of power when it comes to directing access to AT, discouraging participation, limiting interpretation, and enforcing bodily norms. However, where there is power, there is resistance. We observe families assert their own interests and priorities in response to these moments of friction. Sara Ahmed would refer to these moments as experiences of “coming up against” [3]. We conceptualize *minor resistance* (1) as indexing strategies of survival that are everyday and may seem trivial from a politically consequential perspective. While small in scale, minor resistance shows families exercising their agency against oppressive structures through everyday acts of defiance. In this section, we will show the surprising power these “weak” forms of resistance have in building toward an infrastructure of resilience.

#### 5.2.1 Selective compliance.

Selective compliance refers to the different strategies families devise to make themselves legible to external expectations while preserving own way of doing things. In the previous section, we see that “systems” decide whom they choose to listen to when defining what constitutes AT. Oftentimes those definitions do not line up with the families’ own norms and routines, but instead of a direct oppositional stance, families may choose to “acquiesce” on the surface and adapt to external demands. What might appear to be giving in can also be a refusal to assimilate.

For example, P4’s son is 22 years old. He is non-verbal and autistic. For a mix of economic and practical reasons, he has not grown up using a tablet to communicate. He finds iPad unfamiliar and does not like to carry it around with him. Over the years, he has mainly used picture cards and simple sign language to communicate with his family. Since he has mostly encountered iPad in school, when he recently was able to take one home with him for the whole year, he still barely used it. When he is home, he prefers to search for expressions using gestures. His mom says, “*To be honest, I like the effort he makes, trying to tell me things with his mouth and gestures without using the iPad*.” For him, communicating through iPad is more just cumbersome because it takes him a lot of time to search the icons for what he wants to say: “*It’s easier for him if people would understand his gestures*”, says his mom. If they can have things their way, the mom would have preferred a sign language interpreter at school instead of communication tablets. Now at 22 years old, the son has been picking back up the iPad for the past 6 months. Despite the iPad being complicated for him to learn at this age having not grown up with it, they believe it’ll be help his communication with teachers and other children at school. This does not change their daily communication routines at home based in signs and pictures though.

What is notable about this case is that the taking up of AT is not to improve a faulty communication. There is a better and preferred system in place at home, and the use of the tablet is simply to accommodate to others without changing the core dynamics of communication. This can be seen as an act of resistance because it diverts power away their most personal sites such as home. This superficial engagement of power actually lets P4 preserve their existing routines in an unobtrusive way.

When it comes to communicating through the iPad, the goal for many of the families interviewed is not to increase fluency, as is often assumed. Families often use it as a socially responsive tool, as a way for their child to be legible to others, rather than changing how communication happens at home. As P1 puts it, the goal was not for her son to master the iPad per se, but “*more so that he can use it with other people who don’t understand his language…or don’t understand his communication*”. This case illustrates how adoption of an AT can be motivated not by a search for better functional “fit” but by the need to perform social expectations. Importantly, the family does not fully assimilate the iPad into their daily routines, choosing to use it on a as-needed basis rather than making the effort to achieve fluency. For the family, what is useful might look like learning just enough vocabulary and not a full uptake.

Selective compliance is about people intentionally choosing when, where, and how to engage with external norms on their own terms, in ways that prioritize existing linguistic and cultural routines. It is a boundary-setting move in face of pressure to assimilate.

#### 5.2.2 Strategic refusal.

Strategic refusal describes the different ways families opt out of available and seemingly helpful resources, not out of lack of interest, but as a negotiation of what is safe, manageable, and worth the risk in their daily lives. Rather than take up every opportunity for support, families often assess what forms of help may actually come with hidden costs.

One example of this is the disuse of communication tablets (e.g. iPads with communication apps installed). A common reason for disuse is the fear that their child might damage a borrowed device and that they would have to reimburse the organization that lent it to them. For P9, the reluctance to use the school-issued iPad at home was not about the device’s functionality, but the stress that accompanied its use: “*Right now the most important thing here will be the iPad because I think that works very well. If I go purchase that myself,* ***I will be more relaxed****. Because I will see that if [my child] bites that or something happens we will not be scared to help to get charged for that*.” She adds that at home, she only lets her 3-year-old son use the iPad when she’s able to supervise him 100% of the time. Here, institutional support introduces more stress than relief. In this case, the issue isn’t resistance to the device itself, but resistance to the *terms* under which it is offered. This specific fear about damaging devices gestures toward a broader theme: how risk gets factored into families’ decisions about whether and how to engage with ATs.

P6 described a similar calculus in deciding not to borrow a weighted vest from ODMF’s lending library, fearing that forgetting to return it could result in additional costs for the family later on: “*Sometimes when you are a single mom that has other responsibilities and everything going on with the other children, it was just one more thing that I did not want to take care of* ”. Rather than take on that risk, she opted to find solutions “*some other way*”. These workarounds may not promise long-term or optimal outcomes (i.e. trying out different potentially useful devices and getting funding for them), but they grant more control in the moment. As she reflected on the emotional toll of these small decisions accumulating over time, she added, “*It made me not want to ask for more help*”.This line highlights an important insight: for families operating with already stretched resources, help often comes with a price tag (both literally and metaphorically) – guilt if something goes wrong, fear of financial consequences, and exhaustion from navigating complex systems.

This constant tension of juggling competing priorities is particularly salient for P3 whose son has a rare genetic syndrome affecting his mobility. She describes having to weigh every AT consideration against basic needs. When given a list of recommended items from an occupational therapist, she recalled: “*They said, okay, these are the things that you can get. But the only income that I have right now is SSI, so to buy everything that he needs on a regular basis is getting so hard for me*.” Sometimes items such as corrective shoes had to be passed over in favor of food and medication: “*I didn’t get the shoes for $80-$85 if I have to spend on food…I have to manage the money very, very hard*.”

Across these examples, what looks like passive disengagement is in fact a careful and strategic assessment of costs, tradeoffs, and consequences. When the margins are this thin, families become experts in identifying which forms of “help” are truly helpful, and which may ultimately cost more than they give.

On the surface, acts of avoidance may appear passive. But strategic refusal is not about inaction: it is a clear-eyed identification of the sites where power can be exercised. And this awareness is *strategic* precisely because it allows individuals to navigate power dynamics on their own terms.

#### 5.2.3 Covert disobedience.

Covert disobedience describes how families intentionally depart from official guidance – whether from schools, therapists, or healthcare providers – in favor of experience-based knowledge. Compared to previous tactics, this form of disobedience can be seen as the most direct challenge to authority, as it involves a clear departure from the prescribed course of action and a rejection of the belief that professionals always know what’s best. At its core is a conviction that institutional expertise doesn’t always align with lived realities. Parents and caregivers may not always feel empowered to challenge authority directly, but they assert their own judgment in ways that reflect a deep attunement to their child’s needs. These acts of refusal are usually quiet, taking the form of second opinions or parallel paths.

For instance, P10 described how she lost trust in her child’s doctor after being told there was no need for further neurological testing. Rather than accept this guidance, she sought out another provider, one who eventually offered a diagnosis that unlocked access to critical assistive devices at her son’s school.

Similarly, the AT specialist at ODMF shared a case where her client, a mother of a 9-year-old girl with Down Syndrome, disagrees with the treatment plan of the speech therapist. At 9 years old, the child is still unable to form sentences, confuses pronouns, and has difficulty expressing her wants and needs to people outside of her immediate family. She has used an AAC device in the past at school and in speech therapy, and her mother reports seeing a lot of improvement in her verbal communication. However, in the past six months, her mother reports that the speech therapist has stopped using the AAC device, and is “*only working on verbal communication*”. She expressed concerns about discontinuing the use of AAC, because she saw the progress her daughter was making. However, she is reluctant to directly challenge the speech therapist because they are professionals after all. Even so, she is happy to borrow an AAC device from ODMF to use at home. In this way, she created a parallel track, one that honored her own knowledge and observations without direct confrontation.

The impulse to seek alternatives also shows up in how families navigate systemic limitations. One SLP noted that school district-level recommendations are often shaped more by cost and convenience than by individual fit: “*There tends to be, like, one or two types of devices that the school district just picks, usually based on cost. What they recommend for all kids…there are very few kids that get something different from that. So typically it’s an iPad with one of two apps—it’s usually Proloquo or TouchChat*”. Recognizing this one-size-fits-all approach, many families pursue outside AAC assessments to access tools that better reflect their child’s needs:“*Even if the school said, ‘this is the device we recommend’, the families are catching on, that it comes from a pool of maybe one or two [devices]. So they’re also doing their own [assessments], to have a device that’s theirs, that they control, they can edit it and do whatever they want with it, take it wherever they want, but also then have a device that’s prescribed more based on medical and clinical necessity than…a one-size-fits-all approach*”. This workaround is a quieter form of dissent. It challenges not just a particular device recommendation, but also the idea of institutional efficiency. Families are not waiting for school districts to become more flexible – they are finding ways to make things happen for themselves.

These workarounds challenge the idea that institutions are always the best, or only, sources of support. They reflect families’ ongoing efforts to resist being constrained by rigid, often resource-limited infrastructures and instead carve out spaces of autonomy and care. In doing so, families position themselves as experts in their own right. They know how their child communicates, when a device is working, and when a service is falling short. And even when they cannot change the system directly, their quiet deviations reassert the legitimacy of other ways of knowing, other priorities, and other timelines. Covert disobedience, then, is not simply a rejection of authority, it’s an infrastructural critique rendered through practice. It reveals where formal systems fail to flex, and how everyday acts of adaptation and refusal keep families afloat within them.

#### 5.2.4 Folk bridgecraft.

Families often find themselves bridging gaps created by the systems they have to interact with, creating stopgap solutions that give them ad-hoc access to the ATs they need. These gaps might include slow processes, incomplete information, or bureaucratic hurdles, and rather than working within the confines of these systems, families seek temporary solutions to address their immediate needs.

Take P12, a father who had been waiting for months for an approved device to help with his son’s mobility impairment. In the meantime, he adapted an Xbox game controller to build simple games that helped his son exercise his fingers. This makeshift solution not only addressed an immediate need but also is a response to the frustration with a system that was too slow or unresponsive.

Other forms of bridgecraft focus on coordinating or supplementing different forms of AT support. Many parents, faced with gaps in knowledge or experience, end up having to become experts in a variety of topics, from medical information, technology, to insurance policies. P6, for example, described how she needed to become an expert in everything related to her child’s care, including learning about how different ATs worked, sometimes learning relevant medical concepts to understand which treatment plan might work best. She also played the role of communicating between speech therapist and the occupational therapist, ensuring that their recommendations didn’t lead to overlapping AT interventions. With a lack of cross-coordination between the services she and her child encounter, she feels that the burden fell on her to stitch together a care plan that will be most beneficial to her child.

This bridging work often extends into collective action. PM3 emphasized that much of the existing AT support infrastructure, especially for those who fall outside the purview of usual channels for support, is parent-built. She shared: “*That’s why most nonprofits in the disability field are started by parents. [name redacted], for instance, was a mother whose son had autism, English was her second language, and this whole organization started with parents sitting around a kitchen table, sharing needs, identifying gaps in services*.” PM3 explained that formal institutions, especially school systems, tend to offer a narrow set of AT options, usually only for students with very specific needs:

“Because if you have a significant need, or if you’re medically fragile or highly impacted with behavior and disability, your pathway is a bit more supported…and it has a little bit more structure around it. [But] if you’re a young adult who doesn’t qualify for DDA services, but maybe you’re DVR eligible, and you’re a bit more cognitively aware and independent, the path is a lot more murky.”

In these cases, families rely on grassroots groups to share knowledge, swap devices, or navigate informal channels to get what’s needed. These parent-led networks operate as parallel infrastructures, filling in where institutional support stops short.

These examples all reveal a consistent pattern: when formal AT infrastructures are rigid, slow, or out of sync with real needs, families don’t just wait, they create their own solutions. What they build may be temporary or improvised, but it is deeply attuned to their child’s lived context. This is not just a workaround, these acts of bridging are “crip technoscience” [34] in action. By creating their own solutions and pushing the boundaries of available resources, these families are not simply coping with gaps in the system but also actively reshaping the possibilities of what can be done with what’s at hand. The work these families do in these contexts often go unnoticed and undervalued. While professionals might specialize in one area, families integrate knowledge across multiple domains, creating an infrastructure of care and adaptation that exist outside the formal systems, which often becomes a lifeline in the face of structural failure.

## 6 DISCUSSION

Our results show that designing for underserved communities begins with developing a critical consciousness around the *sociotechnical* nature of access work. This is especially important because marginalized communities often have to engage more (not less) with bureaucratic systems like healthcare, welfare, or immigration. As Herd & Moynihan [38] point out, the complexity of government systems (paperwork, eligibility checks, etc.) disproportionately burdens those with fewer resources. Their work, along with our findings, underscore the need for a power-aware perspective in accessibility research and design.

From the perspective of low-resourced contexts, many of the difficulties surrounding AT use – such as navigating fragmented services, encountering institutional gatekeeping, and facing persistent emotional strain – have very little to do with device designs. Rather, they tend to be “wicked” problems [66] entangled with structural inequities that eschew simple solutions. This reframing calls on us to: (1) examine how our own research practices may inadvertently reproduce power imbalances, and (2) recognize communities as potent sites of resistance, innovation, and care – and consider how technology might amplify these grassroots forms of collective action.

### 6.1 Implications for Research

In this section, we pose a series of reflections aimed at helping accessibility researchers critically examine how power operates within the tools and practices of our field. These are meant as entry points for rethinking whose interests are prioritizes and what forms of access are made possible through the principles that guide our design.

#### 6.1.1 AT is not a neutral tool for provisioning accommodation.

The story of P4’s son using the iPad only at school and preferring sign language in all other settings is notable because the communication app on the iPad in his case is not fulfilling a specific need of his; instead, it compensates for the school’s failure to provide a sign language interpreter, which brings to the fore the question: who is the AT helping, anyway? From this case, the method of communication lays bare the power imbalances inherent to the situation. The communication device, as seen in this instance, inscribes power relationships. It constructs P4 as the “other” [84], as different from the allistic instructor, needing to adopt a language that the instructor understands. The story challenges the assumption that AT always acts as an accommodation *for* disabled individual when in reality it sometimes *accommodates* the individual who is unable to provide the appropriate accommodation.

From this case, we can see that AT is not simply a neutral tool that supports access - it is an instrument through which norms about disability are articulated and enforced. The process of seeking and using AT often reveals deeper structures of power: who is recognized as deserving of support, who must justify their needs, and under what terms. Disability scholar Margaret Price [65] has critiqued the individual accommodation model for creating a two-tiered system. Those with more privilege–who can articulate their needs in institutionally legible ways–are more likely to receive accommodations and succeed within dominant systems. Those with less predictable or less socially recognized disabilities are more often excluded.

Instead of understanding AT as a neutral technology, we might consider it as a boundary object [85]. Through this lens, AT becomes a site of negotiation: we are able to see the conflicting interests, tensions, and negotiations that occur between different parties, in this case between P4’s son and the people he encounters at school like his teachers and peers. These moments reveal that access is not just about solving a design problem – it is also about navigating competing definitions of what counts as legitimate need, desirable behavior, or productive participation.

#### 6.1.2 Empowerment means being able to define own AT pathways.

In our study, families made clear that access alone does not guarantee empowerment. The deeper issue is not simply whether services or technologies are available, but whether families are meaningfully supported in exercising their right to choose – on their own terms, and in ways that align with their rhythms, values, and priorities. This aligns with the notion of *power-to*, which emphasizes people’s capacity to act, particularly in contexts where that capacity is constrained by institutional processes and bureaucratic timelines.

Recognizing *power-to* as a core dimension of empowerment for accessibility research invites a shift in how we evaluate success in AT design. Rather than focusing narrowly on whether a device’s features align with a user’s needs, we might instead ask more infrastructural questions [92]: Does this system create the conditions for informed, context-sensitive decision-making? Does it accommodate the “consequence calculus” [54] that disabled people navigate daily? Does it leave room for a range of choices that make sense in the moment, even if those choices fall outside normative expectations of success? Situating empowerment this way shifts design goals that extend beyond usability or adoption, toward infrastructures that lower the cost of choice, respect individual temporalities, and sustain decision-making capacity over time.

#### 6.1.3 Generic recommendations are meaningless without structural context.

Very often, solutions to AT abandonment are proposed without awareness of how they will interact with the systems in which they are meant to be embedded. For example, in AT literature, training is considered a key factor in reducing abandonment [9]. Studies show that when users receive adequate AT training and ongoing support, their likelihood and regularly using AT increases. However, as the findings of this study demonstrate, training and support do not exist in a vacuum. They are mediated by labor, resources, time, and institutional constraints that shape what forms of help are possible, who is expected to provide them, and under what conditions.

For instance, the question of who is responsible for delivering AT training is often left unexamined. Is it the teachers? SLPs? Occupational therapists? As shown in this study, these individuals are already burdened with heavy caseloads and limited support. Recall the SLP who explained that providing families with the documentation necessary for insurance reimbursement, or keeping up with changes in funding processes, requires unpaid labor and continuing education for which they are rarely compensated or supported. These stories illustrate how even well-intentioned solutions can become complicit in maintaining structural inequities if the labor they require is invisibilized or underfunded. Following Liboiron’s notion of compromised agency [49], we argue that the effectiveness of any intervention is shaped not just by the quality of its design but also by the systems of delivery it relies upon. Solutions that appear universally sound – like offering training – are often only viable for those with time and funding to support them.

Thus, recommendations for addressing AT abandonment must be grounded in considerations of the structural conditions under which they will be implemented. Rather than optimizing technologies in isolation, we must account for the institutional and social infrastructures that enable, or inhibit, their sustained use. Without attention to these systemic realities, interventions risk being ineffectual at best, and complicit in reproducing the very barriers they seek to dismantle at worst. This critique extends to academic research, which itself operates within limiting structures. Our study highlights a disconnect between research outputs and the lived realities of families, prompting us to reflect on how to better align our methods, relationships, and timelines with community-engaged visions of accessibility, a question we explore in the next section. As readers engage with the design directions in the next section, we invite them to also hold in mind the ongoing power dynamics and institutional constraints within academia, and to consider what responsibilities researchers have toward the communities our work engages.

### 6.2 Recommendations for Community-driven Design for Accessibility

Minor resistance tactics are creative, situated responses to inaccessible systems. They show how people innovate under constraint, using available materials, networks, and cultural know-how. Each tactic reveals something about the community-derived sensemaking and tacit practices. More than acts of survival, these tactics show patterns of adaptive expertise, situated problem-solving, and relational care. In other words, they point to under-recognized forms of innovation that emerge from below, within communities. Rather than seeing resistance as passive or oppositional, we can see it as a form of grassroots innovation already in motion. A “power-to” design orientation then asks how these strategies might be amplified and supported.

The pedagogy of impossibility [50] invites us to shift the focus of change away from institutions and toward the survival strategies that emerge in their absence. This perspective calls on us to funnel resources to the people and networks already doing the work. In this spirit, we advocate for a model of community-driven innovation in accessibility research, and offer a few sketches of what that could begin to look like.

#### 6.2.1 Building minor resistance into collective actions.

The tactics of minor resistance explored in our study reflect individual families’ responses to systemic inaccess. Yet, these acts rarely happen in isolation. Families often draw on existing care networks (friends, advocates, specialists) to find alternatives and share wisdom. These informal acts of infrastructuring [90] sustain families in the absence of institutional support. But we wonder: how might we build power into these small acts of resistance, so that they can be scaled into grassroots-level collective action?

As argued in Article 9 of the UN convention on the rights of persons with disabilities [57] and elaborated upon in A/HRC/58/33 [58], accessibility is a human right. While state-by-state federally funded protection and advocacy organizations fight for these rights in the United States, where this study took place, they are often focused on the most severe cases (such as the overuse of solitary confinement against disabled individuals [67]). Accessibility research could take up the effort to support grassroots organizing to empower larger systems to change. Research in adjacent fields has explored technology’s ability to support social movements [32, 48], including connecting people to each other and actions they can take [55], supporting people as they collect data [44–46], document problems [26, 27], and studying how they use social media to organize [48]. However, these projects are not typically designed with accessibility in mind, nor are they executed in disability-focused contexts. This presents an opportunity for accessibility researchers to explore how research and design can actively support the work of social movements and to consider the role computing could play in advancing systemic accessibility, perhaps in conversation with Abebe et al.’s [2] call to reimagine the role of computing in social change.

#### 6.2.2 Cataloging as collective memory work.

Across the tactics of minor resistance observed in this study, participants demonstrated deep, situated knowledge about how to navigate complex and often hostile systems. These tactics are informed practices rooted in lived experience, passed along informally through community or trial and error.

This points to cataloging and archiving as important design interventions for preserving and circulating community-based strategies [17]. They can be seen as forms of collective memory work [6]: by documenting informal hacks, locally-repurposed tools, and navigation workarounds, such archives could reduce the epistemic burden on individuals to start from scratch.

The families in our study are often looking for low-cost alternatives to assistive devices that they are unable to obtain due to long waiting time or not having the right assessments. There are already rich, community-driven archives of DIY AT, such as Makers Making Change^1^ (which creates open-source repositories of assistive tech designs that can be locally adapted and 3D-printed), online forums like r/AssistiveTechnology (which include a collection of walkthroughs, product mods, and “hacked” AT solutions not found in clinical literature), or social media accounts like @techowl (which shares everyday mobility-related adaptations). However, these sources are distributed across different platforms and unevenly documented at times. There is a real need for building infrastructures that make community knowledge readily discoverable, shareable and remixable. This might look like building a database of hacks by collating, categorizing, and standardizing existing adaptations that are currently strewn across different blogs, forums, or websites. Such a platform can become a collective resource maintained and grown by the community itself.

#### 6.2.3 Making complex institutional information navigable and investigable.

A recurring theme in participants’ resistance strategies is the immense labor required to make sense of institutional processes, from special government protocols to insurance paperwork and device procurement systems. These processes produce information asymmetry that undermines interpretive agency. People are being asked to make consequential decisions without adequate tools to assess potential outcomes.

To lower the cost of access-related choice, we argue for interfaces that make institutional complexity navigable and investigable. This could perhaps look like the *Usable Privacy Policy Project*^2^ that turns complex web privacy notices into “easy-to-digest format that enables [people] to make more informed privacy decisions as they interact with different websites”. Other formats such as annotated guides or interactive comics [89] could also be explored.

Such systems could also draw inspiration from participatory sensemaking frameworks, like MIT’s Real Talk for Change project^3^, which brings together community members and researchers to interpret complex data and surface shared concerns. Here, the emphasis is on co-producing insight rather than extracting feedback.

Ultimately, to make complexity navigable is to make access a sensemaking practice, honoring the interpretive labor already being done informally in communities, and designing tools that amplify, rather than replace, those efforts.

## 7 CONCLUSION

This paper has examined how power circulates in the everyday lifeworlds of disabled families navigating AT systems. Through ethnographic engagement with families, service providers, and institutional infrastructures, we highlight how decisions around AT use are often shaped by the constraints of chrononormative timelines, institutional protocols, and information opacity. In response, families engage in minor forms of resistance – selective compliance, strategic refusal, covert disobedience, folk bridgecraft – to assert their agency and make space for choices that align with their lived realities.

Rather than treating AT as a neutral tool for accommodation, we argue for an approach to accessibility that is attuned to the politics of infrastructure, the labor of care, and the friction-filled conditions under which access must be negotiated. We offer the notion of *power-to* as a way to expand computing’s role in empowerment – not only to enable independence and interdepence, but also to support the capacity to act on one’s own terms. This reframing opens up design practices that support communities in building grassroots power through relational and self-determined modes of access.

## Figures and Tables

**Table 1:** Overview of minor resistance strategies

Type of Resistance	Trigger / Cause	Power Relation Resisted	Outcome	AT Use Pattern	Tactics
**Selective compliance**	Loss of **definitional agency**	Pressure to assimilate or conform	Preserving existing routines and practices	Intermittent use: “ *Using this for you* ‘	Time-boxing; Place-boxing
**Strategic refusal**	Loss of **assertive agency**	Participation in systems and processes that are costly or harmful	Asserting autonomy over own priorities	Disuse: “*Not using this*”	Opting out; Abandoning; Hedging
**Covert disobedience**	Loss of **definitional agency**	Authoritative claims about what is right or best	Defending lived expertise	Counter-use: “*Using that instead*”	Borrowing; Second opinion
**Folk bridgecraft**	Loss of **interpretive agency**; Loss of **temporal agency**	Knowledge silos and temporal discontinuities produced through organizational structures	Building flexibility into rigid systems	Adaptive use: *Infrastructuring workarounds*	Stopgap solutions; Knowledge brokering; Peer-to-peer support

## CCS CONCEPTS

• Human-centered computing → Accessibility theory, concepts and paradigms; Empirical studies in accessibility.

## Footnotes

1
https://www.makersmakingchange.com/s/

2
https://usableprivacy.org/

3
https://realtalkforchange.org/
